# Dosimetric Validation of Ethos Online Adaptive Radiotherapy for Prostate Cancer: An End-to-End Phantom Study

**DOI:** 10.3390/life16101692

**Published:** 2026-10-09

**Authors:** Marzena Janiszewska, Maciej Raczkowski

**Affiliations:** Department of Medical Physics, Lower Silesian Oncology, Pulmonology and Hematology Center, 53-413 Wroclaw, Poland

**Keywords:** online adaptive radiotherapy, prostate cancer, Ethos, RUBY phantom, end-to-end testing, dosimetric verification, dose–volume histogram, ICRU Report 83, measurement uncertainty

## Abstract

Background/Objectives: Online adaptive radiotherapy (oART) re-optimizes treatment on the anatomy of the day, but automatically generated plans require dosimetric verification and dose–volume assessment. We performed an end-to-end validation of the Varian Ethos workflow for prostate cancer, including cross-adaptation between two phantom anatomies. Methods: A 3 Gy × 20-fraction reference plan was generated for a RUBY phantom. Scheduled, adapted, and cross-adapted plans were evaluated in two prostate, bladder, and rectum configurations. Absolute dose was measured with a Semiflex 3D ionization chamber at the clinical target volume (CTV), rectum, and bladder and compared with Ethos and independent Mobius calculations. Plan quality was assessed from cumulative dose–volume histograms using ICRU 83-oriented descriptors. Results: For scheduled, adapted, and cross-adapted Ethos deliveries, local differences were within 1.0% at the CTV and 3.6% at the rectum. Bladder percentages were larger, but the underlying absolute differences did not exceed 0.048 Gy. When a cross-scheduled plan was deliberately evaluated on the changed anatomy, dose discrepancies of 0.345 Gy per fraction in the rectum and 0.245 Gy in the bladder exceeded the predefined acceptance limits; adaptation reduced the corresponding SF_IS discrepancies to within 0.015 Gy. Adaptation preserved target coverage and reduced rectal dose in same-anatomy comparisons, with small bladder trade-offs. The representative combined standard uncertainty was 2.71%, with an expanded uncertainty of 5.42% (k = 2); these values were used for uncertainty interpretation and not as location-specific acceptance limits. Conclusions: The Ethos workflow delivered accurate target dose and corrected clinically relevant plan–anatomy mismatch. Dose-volume benefits were anatomy- and objective-dependent rather than uniform.

## 1. Introduction

Prostate radiotherapy is technically demanding because high fractional doses and steep dose gradients are delivered in close proximity to the mobile rectum and urinary bladder [1]. Geometric tolerances are therefore stringent, often on the order of 1–2 mm [2], while pelvic anatomy changes between fractions as the bladder and rectum fill and empty and the prostate changes position and shape. Consequently, a plan based on a single pretreatment anatomy may lose dosimetric accuracy during a treatment course, risking target underdosage or excess dose to organs at risk (OARs).

Online adaptive radiotherapy (oART) addresses this problem by re-optimizing the plan on the treatment couch using the anatomy of the day derived from integrated cone-beam computed tomography (CBCT) or magnetic resonance imaging (MRI) [3,4,5]. Matching the dose distribution to current geometry can support smaller margins and improved organ-at-risk (OAR) sparing. These advantages are particularly relevant to hypofractionated and stereotactic body radiotherapy (SBRT), where each fraction carries a high dose and small geometric or dosimetric deviations may have greater clinical consequences [6].

The Ethos platform (Varian Medical Systems, Palo Alto, CA, USA) has contributed to the clinical adoption of CBCT-guided oART by using artificial-intelligence-supported contouring and automated plan generation while the patient remains on the treatment couch [7]. Its time-constrained workflow places particular emphasis on robust commissioning and quality assurance before clinical implementation.

Because the adapted plan is generated automatically and under time pressure, robust quality assurance must address both geometry and dose. Geometric validation confirms that the daily images and structures are suitable for adaptation and includes CBCT reconstruction, coincidence of imaging and treatment isocentres, image-registration precision, autosegmentation accuracy, and positioning reproducibility [7,8]. Dosimetric validation confirms that the calculated and delivered doses agree and includes assessment of the calculation algorithm, reference and adapted plans, re-optimization, high-gradient regions, and accelerator stability [9,10]. In high-dose-per-fraction treatments, even small geometric or dosimetric deviations may have clinically meaningful consequences for adjacent OARs [11].

An end-to-end (E2E) test evaluates the complete chain from CT imaging and treatment planning through CBCT-based adaptation and dose delivery [12]. A dedicated adaptive phantom such as RUBY (PTW, Freiburg, Germany) can reproduce changes in prostate, bladder, and rectal geometry and accommodate an ionization chamber at predefined measurement points. When combined with an independent secondary calculation and a formal uncertainty budget based on the Guide to the Expression of Uncertainty in Measurement (GUM), the test provides a structured assessment of workflow performance [13]. Measurement-uncertainty analysis can also indicate procedural stability in a quality-assurance system [14]. Comprehensive absolute-dose E2E data for the Ethos prostate workflow, particularly including cross-adaptation and independent verification, remain limited.

International reporting and quality-assurance frameworks are available for intensity-modulated radiotherapy, stereotactic treatments, and MRI-guided radiotherapy through ICRU Reports 83, 91, and 97, respectively [15,16,17]. ICRU Report 83 established dose–volume histogram (DVH) reporting as an integral part of IMRT plan evaluation and recommends the near-minimum dose D98%, median dose D50%, and near-maximum dose D2% for target-volume reporting. Together, these descriptors characterize the lower shoulder, central tendency, and upper tail of the target DVH more robustly than a single point dose. Organ-at-risk assessment should likewise use dose–volume quantities selected for the organ and clinical objective. However, no analogous ICRU report currently provides a dedicated, comprehensive framework for CBCT-guided online adaptive radiotherapy. Thus, measurement-based E2E testing combined with standardized DVH descriptors remains important for developing reproducible commissioning and quality-assurance practice for these workflows.

We used an E2E method to validate the absolute dosimetric accuracy of the Ethos online adaptive prostate workflow, with independent cross-verification against Mobius calculations. We pursued four aims. First, we assessed agreement between ionization-chamber measurements and calculated doses for reference, scheduled, and adapted plans at the CTV, rectum, and bladder. Second, we tested a deliberate anatomy-change scenario by cross-adapting the Type A plan to Type B anatomy and the Type B plan to Type A anatomy. Third, we identified the principal sources of measurement uncertainty in high-dose-gradient regions. Fourth, we compared scheduled and adapted DVHs using ICRU 83-oriented target descriptors and organ-at-risk metrics to determine whether re-optimization improved the planned dose distribution and how the result depended on the direction of anatomical change.

## 2. Materials and Methods

### 2.1. Adaptive Radiotherapy System

The study was performed on the Varian Ethos system (version 2.1), which supports online adaptive radiotherapy within a single session: on-couch CBCT imaging, automatic structure segmentation, plan re-optimization, and immediate delivery.

The adaptive workflow implemented at the Lower Silesian Oncology, Pulmonology and Hematology Center in Wrocław follows four stages—imaging, assessment, re-planning, and quality assurance—supported by the following Ethos components:

1. The Ethos treatment planning system (TPS), used to generate the reference volumetric modulated arc therapy (VMAT) or intensity-modulated radiation therapy (IMRT) plan from the planning CT and reference structures.
2. The Ethos HyperSight imaging system, integrated with the treatment unit, which provides high-quality CBCT images with calibrated HU and electron-density values, artifact-correction algorithms, and AI-based synthetic-CT generation.
3. A segmentation-and-propagation module based on deformable image registration, together with an environment for reviewing and manually correcting the propagated target volumes and organs at risk.
4. The automated Ethos Dose Preview Intelligent Optimization Engine (IOE), which orchestrates plan optimization through the Photon Optimization (PO) algorithm to produce clinically acceptable, dosimetrically accurate dose distributions.
5. The Mobius3D system (version 4.0.2; Mobius Medical Systems, Houston, TX, USA), which provides independent dose calculation and verification. Its integration minimizes data-transfer overhead and lets verification run in step with generation of the scheduled and adapted plans.

### 2.2. Phantom and Measurement Setup

Verification was performed with the RUBY phantom (PTW, Freiburg, Germany), a system designed for precise dosimetric measurements in photon radiotherapy and here applied to adaptive radiotherapy. It is made of tissue-equivalent materials whose mass density and radiologic properties match average soft tissue in the megavoltage range, and its modular construction reproduces the anatomic structures relevant to prostate treatment. The phantom contains anthropomorphic bladder, prostate, and rectum structures, three detector boreholes for the Semiflex 3D ionization chamber (31021), and three CT markers that define the effective measurement points. The body is polystyrene (1.05 g/cm^3^), with organ densities of 1.05 g/cm^3^ (bladder) and 1.07 g/cm^3^ (prostate and rectum). It measures 118 × 241.4 × 118 mm (W × D × H) and weighs 2.45 kg with the organ set.

The phantom accepts two interchangeable inserts, Type A and Type B (Figure 1). These configurations reproduce different bladder and rectal filling states and provide the anatomical variation required to test the online adaptive workflow.

The ionization chamber can be positioned at fixed points corresponding to the organs of interest. Measurements used the Semiflex 3D 31021 chamber (PTW, Freiburg, Germany; 0.07 cm^3^ active volume) with a T10008 electrometer (PTW, Freiburg, Germany). Metrological traceability was ensured by cross-calibration against a Farmer 30013 reference chamber (PTW, Freiburg, Germany; 0.6 cm^3^).

### 2.3. End-to-End Procedure

The E2E test covered the full workflow of treatment preparation and delivery: pelvic CT imaging, reference structure definition, reference planning, CBCT-guided online adaptation, treatment delivery, and dose measurement (Figure 2).

#### 2.3.1. CT Imaging

CT scans were acquired according to the clinical pelvic protocol using a 512 × 512 matrix, the iMAR correction algorithm, 0.58 mm in-plane resolution, 0.5 mm slice thickness, and 580 slices. Separate scans were acquired for the Type A and Type B insert configurations.

#### 2.3.2. Segmentation

We attempted autosegmentation according to the institutional workflow, in which CT images are transferred directly from the scanner to MIM Maestro (version 6.4.2; MIM Software Inc., Cleveland, OH, USA). Because the anthropomorphic phantom does not reproduce the full anatomical context of the human pelvis, the automatically generated reference contours of the prostate, bladder, and rectum were not considered sufficiently reliable for use without correction. We therefore contoured the initial reference structures manually in Eclipse (version 18.0.1; Varian Medical Systems, Palo Alto, CA, USA). We defined Body, rectum, urinary bladder, and CTV–prostate, together with auxiliary structures at each measurement position: SF_IS, representing the active chamber volume, and SF_OS, representing the complete chamber volume including its walls. Separate SF_IS and SF_OS structures were created for the CTV, rectum, and bladder locations.

The planning target volume (PTV) was generated from the CTV using anisotropic margins of 6 mm in the left-right and superior-inferior directions and 3 mm in the anterior–posterior direction. During each online adaptive session, the reference structures were propagated to the fractional CBCT and reviewed before plan assessment and re-optimization. When the session phantom configuration matched the reference configuration (Type A-to-Type A or Type B-to-Type B), the propagated structures generally agreed with the phantom geometry and required only minor manual corrections. In the deliberately mismatched cross-adaptation scenarios (Type A-to-Type B and Type B-to-Type A), substantially larger manual corrections were required before structure approval. Thus, the initial reference-segmentation step should be distinguished from online CBCT structure propagation.

#### 2.3.3. Reference Plan

We generated a reference plan of 3 Gy × 20 fractions in Ethos using seven 6 MV IMRT photon beams. Planning objectives included CTV D98% ≥ 95% and, for the PTV, D98% ≥ 95% and D1% ≤ 103%, all at priority P1; rectal and bladder objectives and priorities are listed in Table 1. For each chamber position, we recorded the TPS mean dose in the active chamber volume (SF_IS) and in the complete chamber volume including the walls (SF_OS) (Figure 3). Dose calculations in Ethos were performed using the Acuros XB dose-calculation algorithm, version 1.1.1001.148, with a dose-calculation grid resolution of 2.5 mm.

#### 2.3.4. Adaptive Delivery

Adaptation was based on CBCT images. For each phantom configuration (Type A and Type B), Ethos generated a scheduled plan, defined as the reference plan recalculated on the current anatomy without re-optimization, and an adapted plan, defined as a fully re-optimized plan for the current anatomy. To evaluate a deliberate plan–anatomy mismatch and its correction, we also performed cross-adaptation: the Type A plan was recalculated and adapted on Type B anatomy, and the Type B plan was recalculated and adapted on Type A anatomy. For consistency throughout the point-dose and DVH analyses, the recalculated non-reoptimized plan in these cross-configuration tests is termed the cross-scheduled plan, whereas the re-optimized plan is termed the cross-adapted plan.

#### 2.3.5. Dose Measurement

We measured doses for the reference, scheduled, adapted, cross-scheduled, and cross-adapted plan variants with the Semiflex 3D 31021 chamber at three predefined locations: CTV, rectum (REC), and urinary bladder (BLA). The SF_IS comparison, representing the chamber active volume, was used as the primary TPS-to-measurement comparison. SF_OS was evaluated as a secondary sensitivity measure to assess the effect of averaging the calculated dose over the complete chamber geometry and is reported in the Supplementary Material. Where available, Mobius provided an independent secondary calculation during the adaptive session. For each chamber position and plan variant, three consecutive dose measurements were performed within the same measurement session. The reported measured dose represents the arithmetic mean, and the sample standard deviation (SD; n = 3) is reported in Table 2, Table 3 and Table 4 and Supplementary Table S1. For the scheduled and adapted measurements, the reference-plan dose was calculated separately for each corresponding treatment session. Consequently, the reference-plan values reported in Table 2 and Table 3 are session-specific and are not expected to be identical.

### 2.4. Dose–Volume Histogram Analysis

We compared cumulative DVHs for the scheduled and adapted plans in Type A and Type B and for both cross-evaluation directions. Following the reporting concepts of ICRU Report 83 [15], target assessment used D98%, D50%, and D2% as near-minimum, median, and near-maximum dose descriptors, supplemented by Dmean. D2% denotes the dose received by the hottest 2% of the structure volume and is a relative-volume descriptor; it should not be interpreted as D2cc. For the rectum and urinary bladder, Dmean and D2% were extracted when available. The Eclipse DVHs represented the complete 60 Gy course delivered in 20 fractions of 3 Gy. To align the presentation with chamber results reported per fraction, dose values expressed in Gy were divided by 20, whereas percentage-based metrics and structure volumes were not rescaled. These values are nominal per-fraction rescalings and do not represent deformable dose accumulation or reconstruction of the dose delivered in individual adaptive fractions. Differences were calculated as adapted minus scheduled. Plan interpretation considered target coverage, target near-maximum dose, and OAR sparing together; no single DVH endpoint was used alone to define plan superiority.

### 2.5. Measurement Uncertainty

Dose measurements under phantom conditions are affected by correction, perturbation, calibration, and geometric factors. In accordance with the GUM framework [13], IAEA TRS-398 [18], and AAPM TG-51 [19,20], the absorbed dose was expressed using the corrected mean charge, calibration coefficient, and relevant correction and perturbation factors.

*D* = *M*∙*N_D_*∙*k_p_*_,*T*_∙*k_pol_*∙*k_s_*∙*k_Q_*∙*k_vol_*∙*k_pos_*

where

- *D*—absorbed dose;
- *M*—mean charge value from the measurement series, corrected for background.

Correction factors:

- *k_p,T_*—temperature and pressure correction;
- *k_pol_*—polarity correction;
- *k_s_*—ion recombination correction;
- *k_Q_*—beam-quality correction.

Perturbation and geometric correction factors:

- *k_vol_*—volume-averaging correction factor;
- *k_pos_*—positioning correction factor.

Calibration factor:

- *N_D_*—ionization-chamber calibration coefficient, here determined by cross-calibration of the Semiflex 3D 31021 chamber against the Farmer 30013 reference chamber (*N_cross_*).

The *k_vol_* and *k_pos_* factors were included in the dose-measurement formalism to account for volume averaging and chamber-positioning effects in regions of non-uniform dose. The uncertainties associated with these corrections were also included as separate contributions in the measurement uncertainty budget. The uncertainty budget comprised two groups: metrological components (calibration, readout, and correction factors) and geometric-clinical components (positioning, local dose gradient, definition of SF_IS and SF_OS, and agreement with TPS dose). This structure follows the GUM approach and is consistent with the use of uncertainty analysis to evaluate the stability of accredited linac QA procedure [13,14].

#### 2.5.1. Metrological Components

The metrological components included:

- Readout repeatability evaluated by a GUM Type A statistical analysis;
- Electrometer uncertainty;
- Temperature and pressure correction (*k_p,T_*);
- Polarity correction (*k_pol_*);
- Ion recombination correction (*k_s_*);
- Beam-quality correction (*k_Q_*);
- Uncertainty of cross-calibration of the Semiflex 3D chamber against the Farmer chamber.

Methods for determining *k_p,T_*, *k_pol_*, *k_s_*, and *k_Q_* were based on the TG-51 [19,20] and TRS-398 [18] formalism. Calibration transfer between chambers was performed in accordance with TRS-398 and commissioning practice described in TG-106 [21]. The combined standard uncertainty of the metrological components, estimated by the root-sum-square method, was:

*u_metro_* ≈ 0.82%(1*σ*)

#### 2.5.2. Geometric-Clinical Components

In conventional and stereotactic techniques, geometric contributions often dominate. The following were considered: chamber-positioning uncertainty in the phantom, the local dose gradient, the volume-averaging effect, the difference between the point dose and the mean dose in the structure volume, the influence of segmentation and image registration, and uncertainties related to CT/CBCT imaging and HU-to-density conversion. Positioning in a high-dose-gradient region was described by:

$$u_{\mathrm{pos}}\approx \left|\frac{\mathrm{dD}}{\mathrm{dx}}\right|\cdot u_{x}$$

For inclusion in the relative uncertainty budget, the absolute positioning uncertainty was normalized to the local dose *D*: *u_pos,rel_* = *u_pos_*/*D*, which is equivalent to *u_pos,rel_* ≈ |(1/*D*)(*dD*/*dx*)|*u_x_*.

This approach is consistent with geometric tolerance analyses for IMRT and SBRT in TG-119 [22] and TG-142 [2]. We estimated volume averaging in small fields and steep gradients using TRS-483 [23] and the small-field formalism [24]. We considered the difference between a chamber measurement and the TPS mean dose in a finite volume in accordance with TPS and measurement-based QA guidance [25,26]. Segmentation and contour propagation were considered with reference to TG-132 [27], and CT/CBCT imaging and density conversion with reference to TG-66 [28] and TG-179 [29]. The combined standard uncertainty of the geometric-clinical components was:

*u_geomtr_* ≈ 2.58%

The uncertainty components associated with *k_vol_* and *k_pos_* were evaluated separately and included in the geometric-clinical uncertainty budget, thereby accounting for the uncertainty remaining after application of the corresponding measurement corrections.

Chamber-positioning uncertainty in the local dose gradient provided the dominant contribution.

#### 2.5.3. Combined Uncertainty

The total standard uncertainty was obtained by the root-sum-square combination of metrological and geometric components:

*u_c_* ≈ 2.71%(1*σ*)

The resulting combined standard uncertainty was approximately 2.71% (1σ), and the expanded uncertainty was approximately 5.42% for *k* = 2. The geometric-clinical standard uncertainty of 2.58% represents a single representative estimate for the phantom E2E measurement procedure and was not recalculated separately for each CTV, rectum, and bladder measurement location. Because the chamber-positioning contribution depends on the local dose gradient and local dose level, the 2.71% and 5.42% values should not be interpreted as location-specific uncertainty or acceptance limits.

### 2.6. Acceptance Criteria and Data Analysis

We evaluated formal acceptance using a prescription-normalized signed dose difference, *Δ_Rx_* = 100 × (*D_meas_* − *D_calc_*)/*D_Rx_*, where *D_Rx_* was 3.0 Gy per fraction. We adopted study-specific limits of ±3% for the target and ±5% for OAR measurements, reflecting the wider geometric sensitivity of high-gradient OAR positions [22,26,30]. For descriptive interpretation, we also calculated the local relative difference, *Δ_local_* = 100 × (*D_meas_* − *D_calc_*)/*D_calc_*, and the absolute difference, |*D_meas_* − *D_calc_*|, in Gy. The local percentage was not used alone to judge low-dose bladder measurements because a small absolute difference can produce a large relative value. We interpreted residual differences in the context of the expanded measurement uncertainty but did not use uncertainty as a substitute for the predefined acceptance criteria. Formal pass/fail classification was based exclusively on *Δ_Rx_*. The local relative difference and the absolute dose difference were used for descriptive interpretation and were not themselves acceptance criteria.

## 3. Results

We measured radiotherapy doses for two RUBY phantom configurations, A and B, together with the cross-adaptations from type A to type B and vice versa. For each scenario we evaluated the difference between the measured and calculated dose for the reference, scheduled, and adapted plans, and the effect of the configuration change on the final agreement. We report three measures of this difference: the local percentage difference, the prescription-normalized percentage difference, and the absolute difference. Both the local percentage and the absolute difference are given because a local percentage can be misleading at low measured doses, as at the low-dose bladder points; the prescription-normalized difference expresses each deviation relative to the 3 Gy fractional prescription and provides the basis for the acceptance criteria. In high-dose-gradient regions we additionally considered the chamber volume (0.07 cm^3^), the positioning uncertainty, and the difference between the point dose and the mean dose within a volume.

### 3.1. The Reference Plan Calculation Agreed with Measurement Within Clinical Tolerance

Both percentage and absolute differences are reported, because a relative difference can be misleading when the measured dose is close to zero. For the reference plan, the calculated mean dose in the active volume (SF_IS) and the measured dose agreed closely in the target and rectum, within ±1.3% at the CTV and rectum (Table 2). In the bladder, the percentage difference was larger. In Type A it reached about 9% at a low measured dose (~0.13 Gy), corresponding to a very small absolute difference (~0.01 Gy); in Type B the bladder received a higher dose (~0.49 Gy), where the reference-plan difference was about 25% but represented an absolute difference of ~0.10 Gy. All target and rectum comparisons satisfied the predefined prescription-normalized acceptance criteria of ±3% for the target and ±5% for OAR measurements [2,25,30].

### 3.2. Target and Rectum Agreement Stayed Within Tolerance for the Scheduled Plan

For the scheduled plan, the calculated and measured doses agreed closely at the CTV and rectum in both configurations (Table 2). In SF_IS, local differences were 0.2% (CTV) and 0.1% (rectum) in Type A, and 1.0% and 2.7% in Type B, corresponding to absolute differences in no more than 0.040 Gy. Expressed relative to the fractional prescription, all target and rectal deviations remained below 1.4%. This confirms that the measurement chain was consistent before any adaptation was tested. In the bladder, the local percentage was larger—8.7% in Type A and 10.8% in Type B—but these corresponded to absolute differences in only 0.010 and 0.048 Gy, or 0.35% and 1.59% of the prescribed fractional dose.

Agreement was tightest in the CTV, where the dose is high and uniform, and looser in the organs at risk. This is expected: the organs at risk lie in steep dose-gradient regions, where chamber positioning and volume averaging have more effect. The bladder illustrates why the local percentage should not be read alone. In Type A the very low absorbed dose (~0.13 Gy) amplifies a 0.010 Gy difference into 8.7%, whereas the same difference is 0.35% of prescription. In Type B the bladder received a higher dose (~0.49 Gy), where the local and prescription-normalized values lie closer together.

### 3.3. The Adapted Plan Agreed with Measurement Within Tolerance in Both Configurations

For the adapted plan, the calculated and measured doses agreed closely at the CTV and rectum in both configurations (Table 3). In the active chamber volume, local differences were 0.3% (CTV) and 2.2% (rectum) in Type A, and 0.5% and 3.6% in Type B, corresponding to absolute differences in no more than 0.050 Gy. Expressed relative to the fractional prescription, all target and rectal deviations remained below 1.7%. CTV agreement matched that of the scheduled plan, confirming that re-optimization preserved target accuracy. Agreement at the rectal measurement position was less close than at the CTV, consistent with its location in a steep dose gradient; however, the corresponding prescription-normalized differences remained within the predefined OAR acceptance criterion. In the bladder, the local percentage again reflected the dose level: 8.7% in Type A at a measured dose of 0.130 Gy (0.010 Gy absolute; 0.35% of prescription), whereas in Type B the bladder received a substantially higher dose (0.769 Gy) and the adapted calculation matched it to within 1.4% (0.011 Gy; 0.35% of prescription). Type B is the only configuration where the bladder dose was high enough for the local percentage to be meaningful, and its adaptation improved agreement from 10.8% (scheduled) to 1.4%.

Relative to the scheduled plan, re-optimization preserved target-dose agreement and met the formal criteria at all measured positions. It did not uniformly reduce the local point-dose differences at the organs at risk—some improved and others increased as the high-gradient region shifted relative to the fixed chamber position. These measurements assess local dosimetric agreement; they do not replace dose–volume evaluation of target coverage or OAR sparing.

### 3.4. Adaptation Recovered Clinical Agreement on a Changed Anatomy, Where the Cross-Scheduled Plan Deviated Markedly

Cross-adaptation provided the clearest test of the adaptive workflow—in this scenario, each plan was delivered on the other configuration’s anatomy (Table 4). The cross-adapted plans reproduced the measured dose closely in both directions: local differences were within 1.0% at the CTV and 2.6% at the rectum, and all cross-adapted deviations remained below 1.4% of the fractional prescription. In the bladder, the local percentage was again amplified by the low dose (~0.13 Gy), reaching about 12% in the active volume, but the absolute differences were only 0.014–0.015 Gy, or below 0.5% of prescription. The deliberately mismatched cross-scheduled OAR comparison exceeded the study-specific OAR criterion in the organ most affected by the anatomy change, whereas cross-adaptation restored agreement.

The benefit of adaptation was clearest in the organs at risk, and it appeared in a different organ in each direction. For the Type A plan on the Type B anatomy, the cross-scheduled plan deviated from the measurement by 0.345 Gy in the rectum (−21.2% locally; −11.5% of prescription), whereas the cross-adapted plan agreed with the measurement to within 0.015 Gy for the primary SF_IS comparison (−1.1% locally; −0.5% of prescription).

For the Type B plan on the Type A anatomy, the corresponding failure occurred in the bladder: the cross-scheduled plan deviated by 0.245 Gy (−64.5% locally; −8.2% of prescription), reduced to 0.015 Gy after cross-adaptation for the primary SF_IS comparison. In each direction, the cross-scheduled plan exceeded the study-specific OAR criterion only in the organ most affected by the anatomy change, while the other organ remained within tolerance. Independent Mobius calculations reproduced the direction of both mismatches. The target dose was unaffected in both directions (CTV within 0.8–1.0% locally), so the dosimetric impact of the anatomy change fell predominantly on the organs at risk. These results show directly that re-optimization compensates for the deliberately introduced plan–anatomy mismatch.

### 3.5. Dose–Volume Histogram Comparison

Whereas the chamber measurements assess agreement at three fixed points, the DVH describes the planned dose across the whole structure volume. We compared the scheduled and adapted plans in each configuration, and in both cross-adaptation directions, using the ICRU 83 target descriptors D98%, D50%, and D2% together with organ-at-risk Dmean and D2% (Figure 4 and Figure 5; Table 5, Table 6, Table 7 and Table 8). Differences are given per fraction and, for interpretability, also as a percentage of the 3 Gy fractional prescription.

#### 3.5.1. Adaptation Improved Rectal Sparing While Maintaining Target Coverage in Both Configurations

In Type A, adaptation preserved target coverage: CTV D98% changed by +0.0006 Gy per fraction (+0.02% of prescription) and PTV D98% by −0.0019 Gy per fraction (−0.06%) (Table 5). Rectal Dmean decreased by 0.1103 Gy per fraction (−3.7%) and rectal D2% by 0.2391 Gy per fraction (−8.0%). Bladder Dmean decreased by 0.0168 Gy per fraction (−0.6%), whereas bladder D2% increased slightly by 0.0267 Gy per fraction (+0.9%). This suggests that the corresponding DVH curves showed a clear rectal benefit with essentially unchanged target coverage and a small high-dose bladder trade-off.

In Type B, CTV D98% decreased by 0.0108 Gy per fraction (−0.36%) and PTV D98% by only 0.0004 Gy per fraction (−0.01%) (Table 6). Adaptation reduced rectal Dmean by 0.0974 Gy per fraction (−3.2%) and rectal D2% by 0.1942 Gy per fraction (−6.5%). Bladder Dmean and D2% increased by 0.0484 and 0.0559 Gy per fraction (+1.6% and +1.9%), respectively. This shows that the rectal gain came with a modest redistribution of dose to the bladder, and with a sub-1% reduction in target dose.

#### 3.5.2. Cross-Adaptation Restored Target Coverage with Direction-Dependent Organ-At-Risk Trade-Offs

The A-to-B comparison demonstrated a redistribution driven by the changed anatomy and competing optimization objectives (Table 7). Adaptation increased PTV D98% from 1.5381 to 2.8499 Gy per fraction (+1.3118 Gy per fraction, equivalent to +43.7% of the fractional prescription). Therefore, the cross-scheduled plan had covered only about half of the prescribed dose at the PTV near-minimum (51%), whereas the cross-adapted plan restored it to 95%. CTV coverage was already adequate in both plans (D98% ≥ 96.7% of prescription) and changed by less than 0.1%, indicating that the underdosage was confined to the target periphery rather than its core.

Rectal D2% decreased by 0.2626 Gy per fraction (−8.8% of prescription), although rectal Dmean increased slightly by 0.0065 Gy per fraction (+0.2%). In contrast, bladder Dmean and D2% increased by 0.2197 and 1.9169 Gy per fraction (+7.3% and +63.9% of prescription)—near-maximum for this organ rose from 31% to 95% of the prescribed fractional dose. Thus, the adapted plan corrected severe PTV underdosage and reduced the near-maximum rectal dose, but with a substantially higher bladder near-maximum on the Type B anatomy.

In the B-to-A direction, the target DVH metrics remained closely matched between the cross-scheduled and cross-adapted plans (Table 8). The additional ICRU Report 83 descriptors showed slight increases in CTV D98% (+0.0065 Gy/fraction) and PTV D98% (+0.0299 Gy/fraction), indicating preservation of near-minimum target coverage. Likewise, CTV D2% (+0.0092 Gy/fraction) and PTV D2% (+0.0139 Gy/fraction) increased only marginally, demonstrating that the cross-adapted plan maintained target-dose homogeneity without clinically relevant dose escalation. At the same time, adaptation reduced rectal Dmean by 0.0965 Gy per fraction (−3.2%), whereas rectal D2% increased by 0.0339 Gy per fraction (+1.1% of prescription). Bladder Dmean decreased by 0.3496 Gy per fraction (−11.7%) and bladder D2% decreased by 0.2713 Gy per fraction (−9.0%). Overall, the B-to-A cross-evaluation demonstrated reduced bladder exposure and lower rectal mean dose, with a small increase in rectal near-maximum dose, while preserving target dose coverage and homogeneity.

Taken together, the DVH comparisons show that an adapted plan should not be expected to shift every OAR curve uniformly toward lower dose. In the same-anatomy tests, the dominant benefit was lower rectal dose with preserved target coverage. In the cross-adaptations, the balance between target recovery and OAR sparing depended on whether the anatomy changed from Type A to Type B or from Type B to Type A. Because of these direction-dependent changes, plan quality should be interpreted from the complete set of target and OAR descriptors rather than from a single DVH endpoint.

### 3.6. Independent Mobius Recalculation Confirmed Target-Dose Agreement and Reproduced Both Cross-Adaptation Findings

Mobius provided an independent secondary calculation of the Ethos results. Target-dose agreement was generally close, whereas larger local discrepancies occurred at some OAR positions. The largest occurred at the Type B scheduled bladder position: Ethos SF_IS was 0.440 Gy and Mobius SF_IS was 0.674 Gy, compared with a measured dose of approximately 0.488 Gy. The triplicate chamber readings at this position were 0.488, 0.489, and 0.488 Gy (mean 0.4883 Gy; SD 0.0006 Gy), indicating that the Ethos–Mobius disagreement cannot be attributed to poor repeatability of the chamber measurement. Potential contributors include differences in dose-calculation algorithms, calculation-grid sampling, representation and density assignment of phantom materials, structure/volume definitions, and finite-volume sampling in a steep dose gradient. These factors were not independently isolated; therefore, the specific cause cannot be determined from the present data. Mobius is consequently interpreted as an independent secondary calculation rather than as a reference standard. Mobius also reproduced the direction of both deliberately introduced cross-configuration mismatches.

### 3.7. Interpretation Against Measurement Uncertainty

The local Ethos–measurement differences in the CTV were within 1.3% for all scheduled, adapted, cross-adapted, and same-anatomy reference-plan comparisons. Rectal differences for scheduled, adapted, and cross-adapted plans were within 3.6%, reaching 0.1% and 2.7% for the scheduled plans and 2.2% and 3.6% for the adapted plans in Type A and Type B, respectively. The expanded uncertainty of 5.42% was used as a representative estimate of overall measurement uncertainty and not as a location-specific acceptance threshold. Formal acceptance was assessed using the predefined prescription-normalized criteria, whereas local relative and absolute dose differences were interpreted descriptively, particularly at low-dose and high-gradient OAR positions.

The comparison between SF_IS and SF_OS illustrates the sensitivity of the calculated mean dose to the selected averaging volume. In Type A, the same measured bladder dose differed by 8.7% from the TPS mean dose in SF_IS but by only 0.3% from the TPS mean dose in SF_OS. Because SF_OS includes the chamber walls and does not represent the detector-sensitive volume, this comparison should be interpreted as a sensitivity analysis of dose averaging in a steep dose gradient rather than as an alternative reference representation of chamber response. The same comparison in Type B, where the bladder dose was several times higher, gave 10.8% and 8.4%—far closer together. This contrast also illustrates why low-dose points should be interpreted using the absolute and prescription-normalized differences together with the local percentage difference.

Low-dose bladder points must be interpreted separately. Local percentages of approximately 8–12% in Type A and in both cross-adaptations may appear large when the calculated dose is small, while the associated absolute differences remained approximately 0.010–0.048 Gy and the prescription-normalized criteria were satisfied. By contrast, the cross-scheduled cross-configuration discrepancies in the rectum and bladder substantially exceeded the formal OAR criterion and represent genuine plan–anatomy mismatch rather than measurement noise. The representative 5.42% expanded uncertainty was not used as a location-specific pass/fail limit.

## 4. Discussion

We validated the dosimetric performance of the Varian Ethos online adaptive prostate workflow using a complete phantom-based E2E procedure. The study combined chamber measurements, two anatomical configurations, scheduled and adapted deliveries, deliberate cross-adaptation testing, independent Mobius calculation, a formal uncertainty budget, and DVH-based plan comparison. This design separated residual measurement agreement from the larger discrepancies caused by applying a fixed plan to changed anatomy and linked point-dose verification with evaluation of the complete planned distribution.

The high-dose CTV showed the most consistent agreement. Ethos treatment-plan calculations remained within approximately 1% locally for scheduled, adapted, and cross-adapted plans, and all target comparisons were comfortably within the prescription-normalized ±3% criterion. This supports the conclusion that online re-optimization did not introduce a systematic target-dose error in the evaluated workflow [9,12]. The magnitude of target-dose agreement observed here is consistent with previous Ethos commissioning and patient-specific QA studies, while the present work extends these evaluations by combining chamber-based E2E measurements, deliberate cross-adaptation, independent secondary dose calculation, DVH analysis, and an explicit measurement-uncertainty framework.

OAR measurements were more sensitive to geometry. The rectum lay in a steep dose-gradient region, where a small displacement changes the sampled dose and where a finite-volume chamber averages the local distribution. The Type A bladder showed marked sensitivity to the TPS volume used for dose averaging: the same measurement differed by 8.7% from the mean dose in SF_IS but by only 0.3% from the mean dose in SF_OS. This difference illustrates the effect of the selected calculation volume in a steep dose gradient, although SF_OS should not be regarded as physically equivalent to the chamber sensitive volume. Residual Ethos differences for scheduled, adapted, and cross-adapted rectal measurements remained within 3.6% locally. This pattern is consistent with geometric sensitivity, but the representative 5.42% expanded uncertainty should not be interpreted as a location-specific uncertainty bound.

The bladder illustrates why local percentages and absolute differences should be reported together. In Type A, the chamber measured approximately 0.13 Gy; consequently, differences of about 0.01 Gy produced local percentages near 8–12%. In the Type B adapted plan, the higher bladder dose allowed a more stable relative comparison, and Ethos agreed within 1.4% locally. Formal acceptance based on the prescribed dose avoided overinterpreting small absolute discrepancies at low-dose points [26].

The cross-adaptation experiment provided the strongest evidence for the dosimetric value of adaptation. In each direction, the cross-scheduled plan exceeded the study-specific OAR criterion in the organ most affected by the anatomy change, whereas re-optimization restored agreement while maintaining the CTV dose. The effect appeared in different organs in the two directions, showing that the relevant failure mode was anatomy-dependent rather than a fixed bias in one detector position or calculation algorithm [3,31].

The DVH results add an important distinction to the point-dose findings. In Type A and Type B, where scheduled and adapted plans were evaluated on the same configuration, re-optimization preserved D98% target coverage and reduced both rectal Dmean and D2%. In Type B this came with a small uniform reduction in target dose—below 1% of prescription across all CTV and PTV descriptors—traded against a 3.2% reduction in rectal mean dose. This shows that adaptation may improve the planned dose distribution even without a deliberate cross-adaptation anatomy mismatch, because the optimizer can redistribute fluence against the current contours and planning priorities [7]. The bladder results also show why this conclusion must remain multi-parametric: some mean or near-maximum bladder doses increased slightly while the rectal endpoints improved.

The directional asymmetry of the cross-adaptation is clinically and physically plausible. A-to-B and B-to-A are not inverse numerical tests, because the relative positions and volumes of the target, rectum, and bladder differ and the optimizer resolves different competing objectives in each anatomy. In A-to-B, adaptation recovered PTV D98% and reduced rectal D2% but increased bladder exposure substantially, with bladder D2% rising from 31% to 95% of the prescribed fractional dose. In B-to-A, adaptation reduced rectal Dmean and both bladder Dmean and D2%, while rectal D2% increased slightly, with target coverage preserved. Thus, the benefit of adaptation was anatomy- and objective-dependent rather than a universal downward displacement of every OAR DVH.

The ICRU 83 reporting framework [15] was useful for structuring this interpretation. D98%, D50%, and D2% sample the lower shoulder, central region, and upper tail of the target DVH, respectively, and reveal different failure modes that a single mean or point dose can miss. In particular, the severe scheduled PTV D98% loss in the A-to-B comparison was evident despite similar CTV and median target doses. Combining these target descriptors with OAR Dmean and D2% made the optimization trade-offs explicit and prevented classification of one plan as globally superior on the basis of a single curve.

Independent Mobius calculations supported the target-dose findings and reproduced the direction of both cross-adaptation mismatches. The largest local Mobius discrepancy occurred at the Type B scheduled bladder position, where Mobius SF_IS was 0.674 Gy versus 0.440 Gy for Ethos and approximately 0.488 Gy measured. Because the triplicate measurement at this position was highly repeatable (SD 0.0006 Gy), readout variability is unlikely to explain the discrepancy. Plausible contributors include differences in dose-calculation algorithms, calculation-grid sampling, phantom-material density representation, structure/volume definitions, and finite-volume averaging in a steep gradient. The experiment did not isolate these contributions, so no single mechanism can be assigned.

This study has several limitations. Because we used a single RUBY phantom with two predefined static anatomical configurations, we did not reproduce inter- or intrafraction motion, patient-specific heterogeneity, or the full complexity of clinical anatomy. Chamber measurements were obtained at three positions and did not validate the complete three-dimensional dose distribution experimentally. The triplicate chamber measurements characterize within-session measurement repeatability but do not quantify between-session or day-to-day reproducibility of the complete CBCT-to-adaptation-to-delivery chain. The DVHs describe TPS-planned dose distributions; their division by 20 provides a nominal per-fraction presentation but is not deformable dose accumulation or reconstruction of delivered adaptive fractions. Initial reference autosegmentation was not sufficiently reliable for the phantom and therefore required manual delineation. In contrast, online structure propagation remained usable: only minor manual corrections were generally required for matched Type A-to-Type A and Type B-to-Type B configurations, whereas substantially greater corrections were necessary in the deliberately mismatched Type A-to-Type B and Type B-to-Type A scenarios. These observations should not be interpreted as validation of clinical AI-based autosegmentation. Overall, the results should be interpreted as commissioning-style E2E evidence rather than as characterization of the full range of anatomical variability encountered clinically.

The tested prescription was 3 Gy × 20 fractions. The E2E concept and uncertainty framework are transferable to other fractionation schedules, but these data do not independently validate stereotactic dose-per-fraction delivery [30]. Each prescription, planning technique, and clinical workflow should undergo dedicated commissioning and verification under the relevant delivery conditions.

Within these limitations, the results support the dosimetric validity of the evaluated CBCT-guided oART workflow. They also show that prescription-normalized acceptance criteria, local relative and absolute point-dose differences, measurement uncertainty, and DVH descriptors answer complementary questions. For a routine clinical use, a robust adaptive-plan assessment should combine delivery verification with multi-parameter evaluation of target coverage and anatomy-specific OAR trade-offs.

## 5. Conclusions

The Varian Ethos online adaptive workflow delivered accurate target dose in this prostate E2E phantom study. Scheduled, adapted, and cross-adapted Ethos plans met the study-specific prescription-normalized criteria, with local CTV differences within approximately 1% and rectal differences within 3.6%. The representative combined standard uncertainty was 2.71%, and the expanded uncertainty was 5.42% (k = 2); these values were used for uncertainty interpretation and not as location-specific acceptance limits.

The clearest evidence for adaptation came from the cross-adaptation test. When the cross-scheduled plan was deliberately evaluated on the alternate anatomy, the dose discrepancy exceeded the predefined acceptance criteria in both cases, whereas re-optimization reduced the corresponding SF_IS differences to within 0.015 Gy.

DVH analysis showed that adaptation can improve rectal sparing while preserving target coverage even in same-configuration comparisons, although modest bladder trade-offs may occur. Cross-adaptation demonstrated the practical value of re-optimization and the direction-dependent nature of its benefit: the Type A-to-B adaptation restored PTV coverage and reduced rectal near-maximum dose, whereas the Type B-to-A adaptation reduced rectal mean dose and both bladder mean and near-maximum dose, with a small increase in rectal near-maximum dose, while preserving target coverage and dose homogeneity, as confirmed by D98%, D50%, D2%, and Dmean. Consequently, adaptive plan quality should be judged from the combined ICRU-oriented target descriptors and organ-at-risk metrics, not from a single DVH curve or point measurement.

Within the limits of a phantom-based test, these results support the dosimetric validity of the evaluated CBCT-guided adaptive workflow, and the E2E phantom approach provides a practical method for commissioning, quality assurance, and further clinical validation.

## Figures and Tables

**Figure 1:**
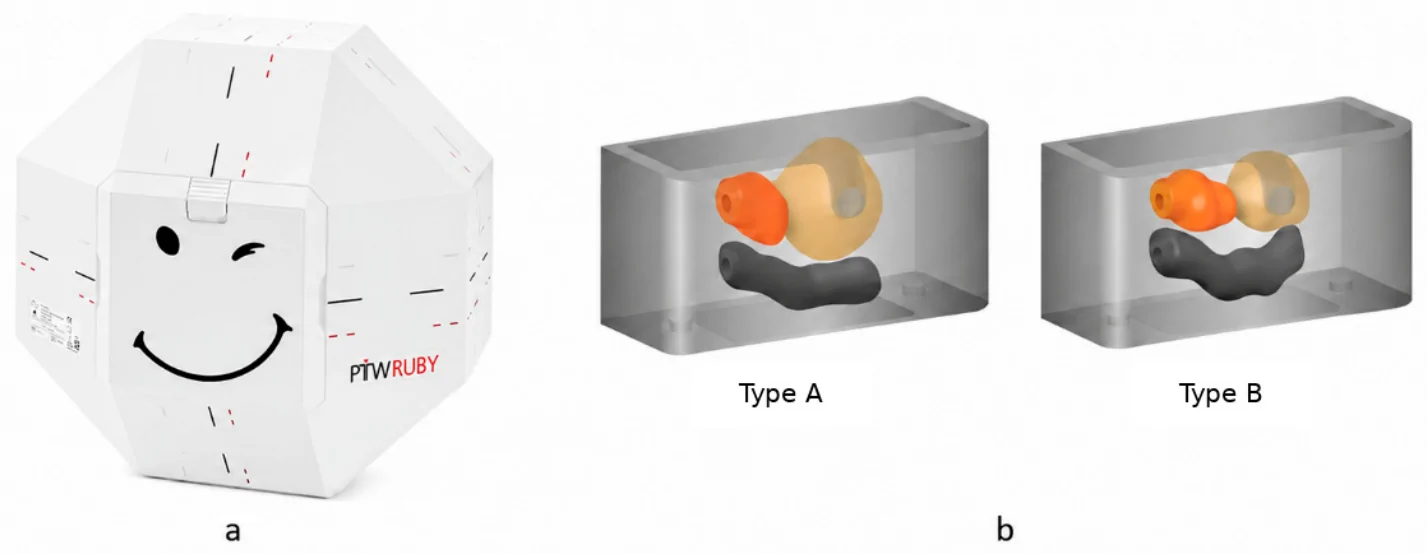
(**a**) Phantom for E2E adaptive tests. (**b**) RUBY Type A and Type B organ sets. Orange indicates the prostate, yellow the urinary bladder, and dark gray the rectum.

**Figure 2:**
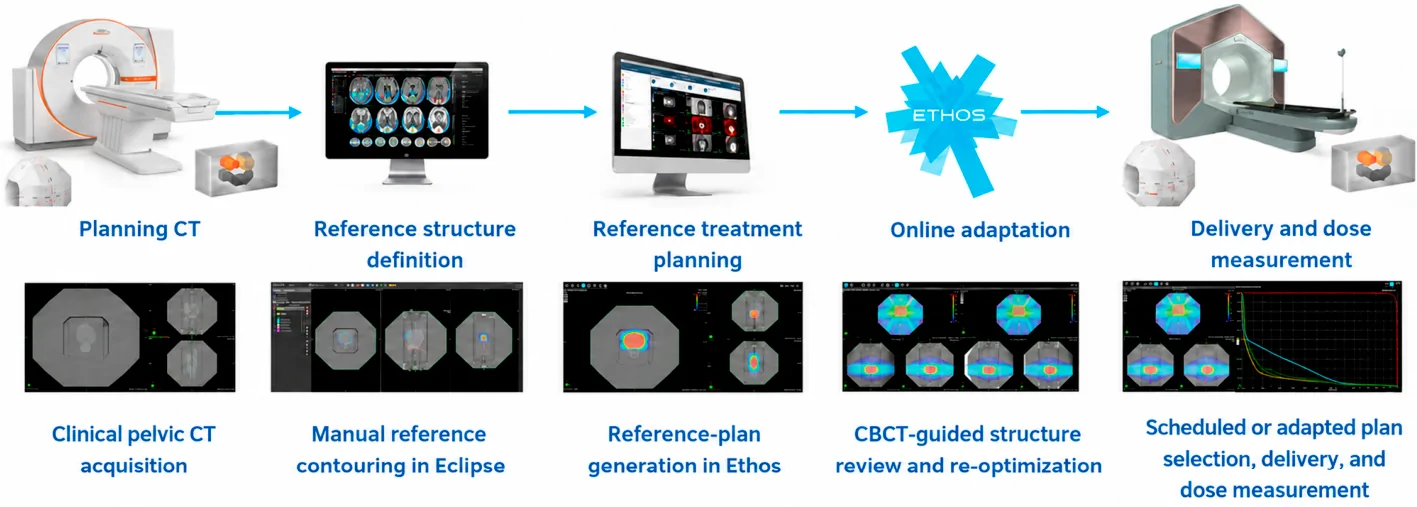
End-to-end workflow for the prostate/pelvis phantom study: planning CT acquisition, manual definition of reference structures in Eclipse, reference treatment planning in Ethos, CBCT-guided online adaptation, plan selection, delivery, and dose measurement.

**Figure 3:**
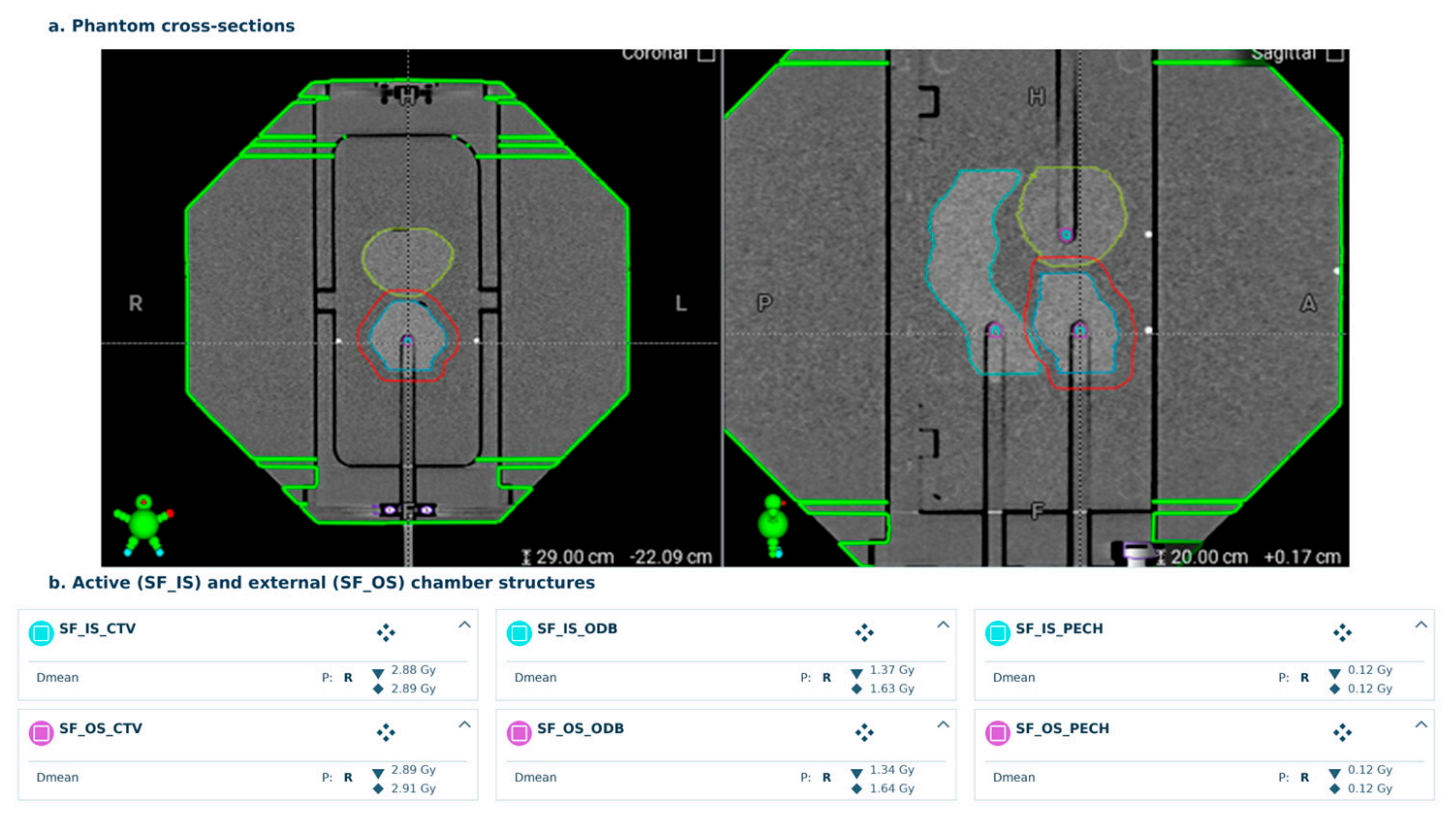
RUBY phantom screenshots: (**a**) coronal and sagittal views; (**b**) Semiflex 3D 31021 chamber structures. Turquoise icons denote SF_IS (active chamber volume); purple icons denote SF_OS (complete chamber volume, including walls). ODB and PECH correspond to rectum (REC) and bladder (BLA), respectively.

**Figure 4:**
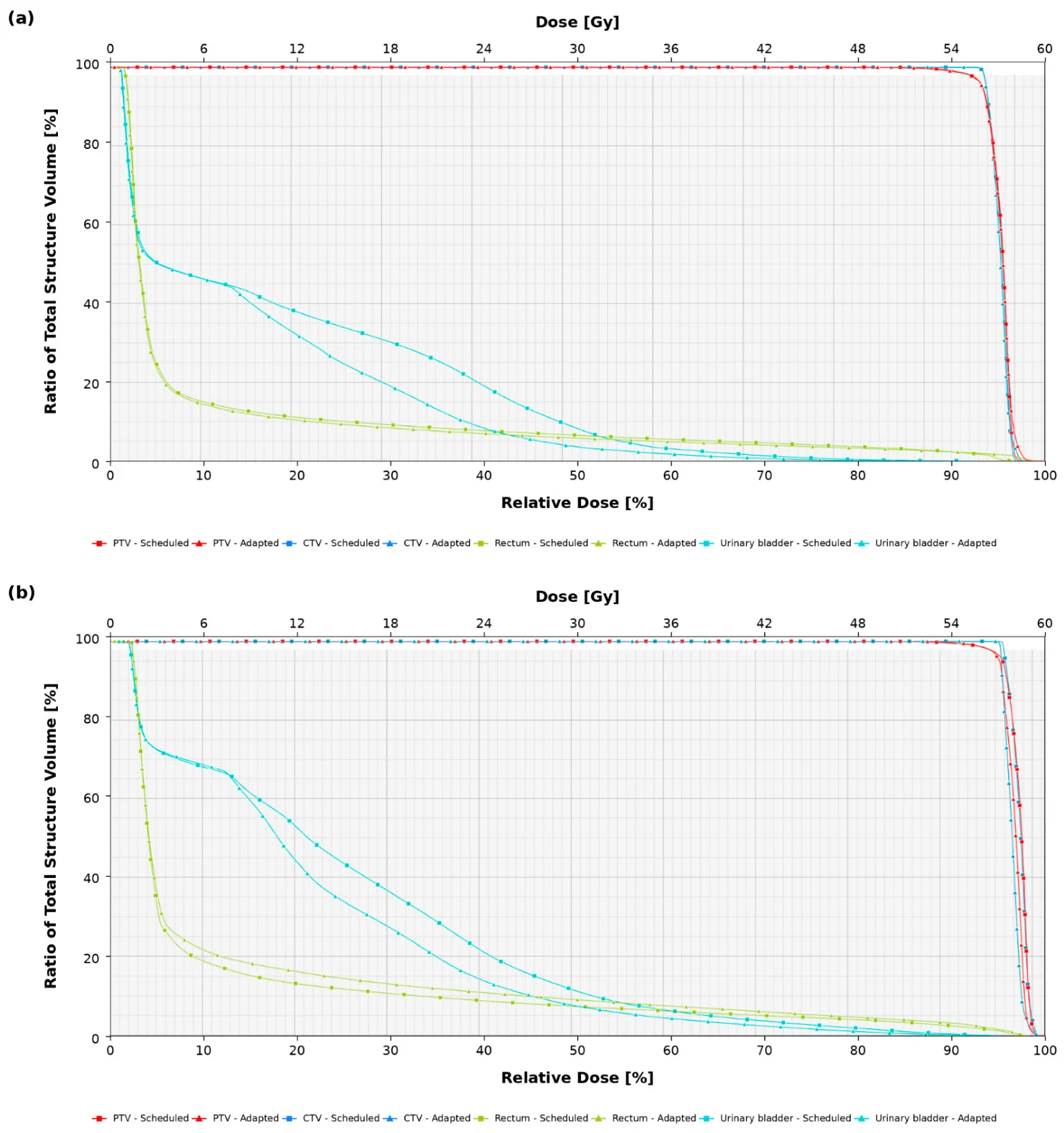
Cumulative dose–volume histograms comparing the scheduled plan (square markers) and adapted plan (triangular markers) for (**a**) Type A and (**b**) Type B. Line colors correspond to the Eclipse structure colors for CTV, PTV, rectum, and urinary bladder. The horizontal axis shows cumulative course dose (60 Gy in 20 fractions); Table 5 and Table 6 report nominal per-fraction values.

**Figure 5:**
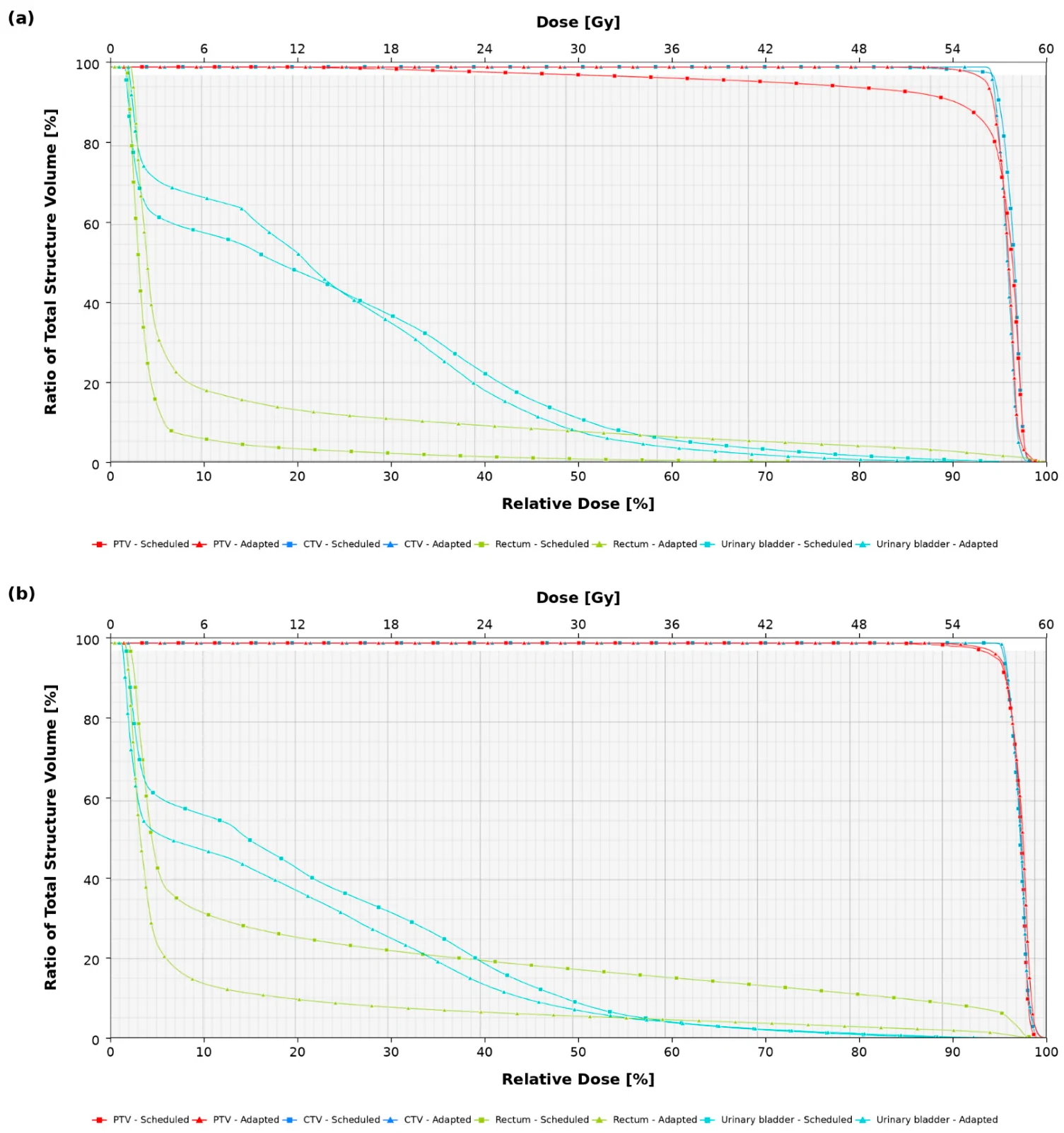
Cumulative dose–volume histograms comparing cross-scheduled (square markers) and cross-adapted (triangular markers) plans for (**a**) the Type A plan evaluated on Type B anatomy and (**b**) the Type B plan evaluated on Type A anatomy. The direction-dependent differences reflect the changed organ geometry and the trade-offs imposed by target and OAR optimization objectives.

**Table 1:** Treatment-plan dose objectives. Var denotes acceptable variation.

Structure	Meaning	Phase	Type	Parameter	Operator	Value	Unit	Priority
Bladder	Urinary bladder	1	Goal	V30.00	<	50	%	P:2
Bladder	Urinary bladder	1	Var	V30.00	≤	65	%	
Bladder	Urinary bladder	1	Goal	V40.00	<	35	%	P:2
Bladder	Urinary bladder	1	Var	V40.00	≤	50	%	
Bladder	Urinary bladder	1	Goal	V50.00	<	25	%	P:2
Bladder	Urinary bladder	1	Var	V50.00	≤	30	%	
Bladder	Urinary bladder	1	Goal	V60.00	≤	5	%	P:2
Bladder	Urinary bladder	1	Var	V60.00	≤	10	%	
Bladder	Urinary bladder	1	Goal	D1.0	≤	65	Gy	P:2
Bladder	Urinary bladder	1	Var	D1.0	≤	70	Gy	
Bladder	Urinary bladder	1	Goal	V48.00	≤	25	%	P:2
Bladder	Urinary bladder	1	Var	V48.00	≤	30	%	
Rectum	Rectum	1	Goal	V20.00	<	85	%	P:2
Rectum	Rectum	1	Goal	V30.00	<	57	%	P:2
Rectum	Rectum	1	Var	V30.00	≤	70	%	
Rectum	Rectum	1	Goal	V40.00	<	38	%	P:2
Rectum	Rectum	1	Var	V40.00	≤	60	%	
Rectum	Rectum	1	Goal	V50.00	<	22	%	P:2
Rectum	Rectum	1	Var	V50.00	≤	40	%	
Rectum	Rectum	1	Goal	V60.00	≤	1	%	P:1
Rectum	Rectum	1	Var	V60.00	≤	3	%	
Rectum	Rectum	1	Goal	D1.0	≤	65	Gy	P:2
Rectum	Rectum	1	Var	D1.0	≤	70	Gy	

**Table 2:** Scheduled- and reference-plan dose comparisons for Type A and Type B configurations using SF_IS. Measured dose is reported as mean ± sample SD (n = 3). SF_OS results are provided in Supplementary Table S2.

Configuration	Plan	Location	Dose [Gy]			Ethos vs. Measurement			Mobius vs. Measurement	
			Ethos SF_IS	Mobius SF_IS	Measured Mean ± SD	Ethos Local Δ [%]	Ethos ΔRx [%]	Ethos |Δ| [Gy]	Mobius Local Δ [%]	Mobius ΔRx [%]
Type A	Scheduled	CTV	2.900	2.939	2.9063 ± 0.0015	+0.2	+0.21	0.006	−1.1	−1.08
Type A	Reference	CTV	2.900	2.955	2.9063 ± 0.0015	+0.2	+0.21	0.006	−1.7	−1.63
Type A	Scheduled	Rectum	1.670	1.621	1.6720 ± 0.0010	+0.1	+0.08	0.002	+3.1	+1.69
Type A	Reference	Rectum	1.670	1.639	1.6720 ± 0.0010	+0.1	+0.08	0.002	+2.0	+1.10
Type A	Scheduled	Bladder	0.120	0.103	0.1303 ± 0.0012	+8.7	+0.35	0.010	+26.8	+0.92
Type A	Reference	Bladder	0.120	0.101	0.1303 ± 0.0012	+8.7	+0.35	0.010	+29.2	+0.98
Type B	Scheduled	CTV	2.910	2.939	2.9380 ± 0.0010	+1.0	+0.93	0.028	−0.0	−0.04
Type B	Reference	CTV	2.900	2.941	2.9380 ± 0.0010	+1.3	+1.27	0.038	−0.1	−0.09
Type B	Scheduled	Rectum	1.450	1.419	1.4900 ± 0.0017	+2.7	+1.32	0.040	+5.0	+2.37
Type B	Reference	Rectum	1.470	1.456	1.4900 ± 0.0017	+1.3	+0.65	0.020	+2.3	+1.11
Type B	Scheduled	Bladder	0.440	0.674	0.4883 ± 0.0006	+10.8	+1.59	0.048	−27.7	−6.22
Type B	Reference	Bladder	0.390	0.622	0.4883 ± 0.0006	+25.1	+3.26	0.098	−21.6	−4.48

Note: Local difference = 100 × (Dmeas − Dcalc)/Dcalc; Rx-normalized difference = 100 × (Dmeas − Dcalc)/3.0 Gy; absolute difference = |Dmeas − Dcalc|. SF_IS denotes the active chamber volume.

**Table 3:** Adapted- and reference-plan dose comparisons for Type A and Type B configurations using SF_IS. Measured dose is reported as mean ± sample SD (n = 3). SF_OS results are provided in Supplementary Table S3.

Configuration	Plan	Location	Dose [Gy]			Ethos vs. Measurement			Mobius vs. Measurement	
			Ethos SF_IS	Mobius SF_IS	Measured Mean ± SD	Ethos Local Δ [%]	Ethos ΔRx [%]	Ethos |Δ| [Gy]	Mobius Local Δ [%]	Mobius ΔRx [%]
Type A	Adapted	CTV	2.910	2.931	2.9180 ± 0.0010	+0.3	+0.27	0.008	−0.5	−0.44
Type A	Reference	CTV	2.900	2.955	2.9180 ± 0.0010	+0.6	+0.60	0.018	−1.3	−1.24
Type A	Adapted	Rectum	1.410	1.399	1.4413 ± 0.0006	+2.2	+1.05	0.031	+3.1	+1.43
Type A	Reference	Rectum	1.480	1.486	1.4413 ± 0.0006	−2.6	−1.29	0.039	−3.0	−1.50
Type A	Adapted	Bladder	0.120	0.108	0.1303 ± 0.0006	+8.7	+0.35	0.010	+20.8	+0.75
Type A	Reference	Bladder	0.120	0.101	0.1303 ± 0.0006	+8.7	+0.35	0.010	+29.2	+0.98
Type B	Adapted	CTV	2.900	2.924	2.9153 ± 0.0006	+0.5	+0.50	0.015	−0.3	−0.30
Type B	Reference	CTV	2.900	2.941	2.9153 ± 0.0006	+0.5	+0.50	0.015	−0.9	−0.85
Type B	Adapted	Rectum	1.380	1.292	1.4307 ± 0.0006	+3.6	+1.66	0.050	+10.7	+4.60
Type B	Reference	Rectum	1.470	1.456	1.4307 ± 0.0006	−2.7	−1.34	0.040	−1.8	−0.89
Type B	Adapted	Bladder	0.780	0.934	0.7693 ± 0.0006	−1.4	−0.35	0.011	−17.6	−5.49
Type B	Reference	Bladder	0.680	0.622	0.7693 ± 0.0006	+13.1	+2.98	0.089	+23.7	+4.91

Note: Local difference = 100 × (Dmeas − Dcalc)/Dcalc; Rx-normalized difference = 100 × (Dmeas − Dcalc)/3.0 Gy; absolute difference = |Dmeas − Dcalc|. SF_IS denotes the active chamber volume.

**Table 4:** Cross-scheduled and cross-adapted dose comparisons for the Type A-to-Type B and Type B-to-Type A cross-evaluations using SF_IS. Measured dose is reported as mean ± sample SD (n = 3). SF_OS results are provided in Supplementary Table S4.

Configuration	Plan	Location	Dose [Gy]			Ethos vs. Measurement			Mobius vs. Measurement	
			Ethos SF_IS	Mobius SF_IS	Measured Mean ± SD	Ethos local Δ [%]	Ethos ΔRx [%]	Ethos |Δ| [Gy]	Mobius Local Δ [%]	Mobius ΔRx [%]
A→B	Cross-adapted	CTV	2.900	2.943	2.9283 ± 0.0006	+1.0	+0.93	0.028	−0.5	−0.50
A→B	Cross-scheduled	CTV	2.890	2.944	2.9283 ± 0.0006	+1.3	+1.27	0.038	−0.5	−0.53
A→B	Cross-adapted	Rectum	1.300	1.350	1.2847 ± 0.0006	−1.1	−0.50	0.015	−4.8	−2.16
A→B	Cross-scheduled	Rectum	1.630	1.639	1.2847 ± 0.0006	−21.2	−11.50	0.345	−21.6	−11.81
A→B	Cross-adapted	Bladder	0.120	0.113	0.1340 ± 0.0010	+12.0	+0.48	0.014	+19.4	+0.73
A→B	Cross-scheduled	Bladder	0.120	0.107	0.1340 ± 0.0010	+12.0	+0.48	0.014	+26.1	+0.93
B→A	Cross-adapted	CTV	2.900	2.925	2.9217 ± 0.0006	+0.8	+0.74	0.022	−0.1	−0.09
B→A	Cross-scheduled	CTV	2.900	2.941	2.9217 ± 0.0006	+0.8	+0.74	0.022	−0.6	−0.62
B→A	Cross-adapted	Rectum	1.530	1.500	1.5693 ± 0.0012	+2.6	+1.31	0.039	+4.6	+2.32
B→A	Cross-scheduled	Rectum	1.470	1.456	1.5693 ± 0.0012	+6.8	+3.31	0.099	+7.8	+3.76
B→A	Cross-adapted	Bladder	0.120	0.113	0.1347 ± 0.0006	+12.3	+0.49	0.015	+19.8	+0.74
B→A	Cross-scheduled	Bladder	0.380	0.322	0.1347 ± 0.0006	−64.5	−8.17	0.245	−58.1	−6.24

Note: Local difference = 100 × (Dmeas − Dcalc)/Dcalc; Rx-normalized difference = 100 × (Dmeas − Dcalc)/3.0 Gy; absolute difference = |Dmeas − Dcalc|. SF_IS denotes the active chamber volume.

**Table 5:** Per-fraction DVH parameters for the scheduled and adapted plans in Type A.

Structure	DVH Parameter	Scheduled Plan (Gy/Fraction)	Adapted Plan (Gy/Fraction)	Difference: Adapted − Scheduled (Gy/Fraction)
CTV	D98%	2.8985	2.8990	+0.0006
	D50%	2.9595	2.9546	−0.0050
	D2%	2.9944	3.0081	+0.0137
	Dmean	2.9541	2.9518	−0.0023
PTV	D98%	2.8540	2.8521	−0.0019
	D50%	2.9630	2.9627	−0.0003
	D2%	2.9992	3.0211	+0.0220
	Dmean	2.9538	2.9545	+0.0007
Rectum	Dmean	0.5679	0.4576	−0.1103
	D2%	2.0366	1.7975	−0.2391
Urinary bladder	Dmean	0.3113	0.2946	−0.0168
	D2%	2.8427	2.8694	+0.0267

Note: D98% and D2% denote the doses received by at least 98% and 2% of the structure volume, respectively; D50% is the median dose; Dmean is the mean dose. Difference = adapted − scheduled. Values are nominal physical doses per fraction obtained by dividing the cumulative 20-fraction DVH values by 20; this rescaling is not deformable dose accumulation.

**Table 6:** Per-fraction DVH parameters for the scheduled and adapted plans in Type B.

Structure	DVH Parameter	Scheduled Plan (Gy/Fraction)	Adapted Plan (Gy/Fraction)	Difference: Adapted − Scheduled (Gy/Fraction)
CTV	D98%	2.9079	2.8971	−0.0108
	D50%	2.9636	2.9356	−0.0281
	D2%	3.0076	2.9931	−0.0145
	Dmean	2.9598	2.9359	−0.0239
PTV	D98%	2.8589	2.8585	−0.0004
	D50%	2.9670	2.9478	−0.0192
	D2%	3.0026	2.9906	−0.0121
	Dmean	2.9578	2.9416	−0.0162
Rectum	Dmean	0.7464	0.6490	−0.0974
	D2%	2.3966	2.2024	−0.1942
Urinary bladder	Dmean	0.3571	0.4055	+0.0484
	D2%	2.7936	2.8495	+0.0559

Note: D98% and D2% denote the doses received by at least 98% and 2% of the structure volume, respectively; D50% is the median dose; Dmean is the mean dose. Difference = adapted − scheduled. Values are nominal physical doses per fraction obtained by dividing the cumulative 20-fraction DVH values by 20; this rescaling is not deformable dose accumulation.

**Table 7:** Per-fraction DVH parameters for the Type A-to-Type B cross-adaptation (cross-scheduled versus cross-adapted plan).

Structure	DVH Parameter	Cross-Scheduled Plan (Gy/Fraction)	Cross-Adapted Plan (Gy/Fraction)	Difference: Cross-Adapted − Cross-Scheduled (Gy/Fraction)
CTV	D98%	2.9035	2.9014	−0.0021
	D50%	2.9766	2.9539	−0.0227
	D2%	3.0128	2.9996	−0.0133
	Dmean	2.9685	2.9521	−0.0164
PTV	D98%	1.5381	2.8499	+1.3118
	D50%	2.9701	2.9578	−0.0124
	D2%	3.0147	3.0171	+0.0024
	Dmean	2.8856	2.9512	+0.0656
Rectum	Dmean	0.7011	0.7076	+0.0065
	D2%	2.3390	2.0765	−0.2626
Urinary bladder	Dmean	0.1417	0.3614	+0.2197
	D2%	0.9291	2.8460	+1.9169

Note: D98% is the near-minimum dose, D50% is the median dose, D2% is the near-maximum dose, and Dmean is the mean dose. Difference = cross-adapted − cross-scheduled. Values are nominal physical doses per fraction derived from the cumulative 20-fraction DVH.

**Table 8:** Per-fraction DVH parameters for the Type B-to-Type A cross-adaptation (cross-scheduled versus cross-adapted plan).

Structure	DVH Parameter	Cross-Scheduled Plan (Gy/Fraction)	Cross-Adapted Plan (Gy/Fraction)	Difference: Cross-Adapted − Cross-Scheduled (Gy/Fraction)
CTV	D98%	2.8950	2.9015	+0.0065
	D50%	2.9531	2.9561	+0.0030
	D2%	2.9963	3.0055	+0.0092
	Dmean	2.9498	2.9522	+0.0025
PTV	D98%	2.8281	2.8580	+0.0299
	D50%	2.9574	2.9624	+0.0050
	D2%	2.9902	3.0041	+0.0139
	Dmean	2.9466	2.9535	+0.0069
Rectum	Dmean	0.6270	0.5306	−0.0965
	D2%	2.1194	2.1533	+0.0339
Urinary bladder	Dmean	0.6272	0.2777	−0.3496
	D2%	2.9530	2.6817	−0.2713

Note: D98% represents the near-minimum dose received by 98% of the target volume, whereas D2% represents the near-maximum dose delivered to 2% of the target volume, in accordance with ICRU Report 83. Together with D50% and Dmean, these parameters provide a more comprehensive assessment of target dose coverage and dose homogeneity in the B-to-A cross-evaluation. Per-fraction values were obtained by dividing the cumulative DVH dose values by 20 and represent nominal physical dose per fraction rather than deformably accumulated delivered dose.

## Data Availability

The data supporting the findings of this study are included in the article and its Supplementary Materials. Additional data are available from the corresponding author upon reasonable request.

## Supplementary Materials

The following supporting information can be downloaded at: https://www.mdpi.com/article/10.3390/life16101692/s1, Table S1: Repeatability of triplicate ion-chamber measurements; Table S2: SF_OS sensitivity analysis corresponding to scheduled/reference-plan measurements; Table S3: SF_OS sensitivity analysis corresponding to adapted/reference-plan measurements; Table S4: SF_OS sensitivity analysis corresponding to cross-evaluation measurements.
