# Supplementary material for: Dosimetric Validation of Ethos Online Adaptive Radiotherapy for Prostate Cancer: An End-to-End Phantom Study
Source: Life (Basel). 2026 Oct 9;16(10):1692. doi: 10.3390/life16101692 (PMC13706040; doi:10.3390/life16101692)
Supplement: Supplementary file 1 [file life-16-01692-s001.zip › life-4542519-supplementary.pdf]

# Supplementary Material

Dosimetric Validation of Ethos Online Adaptive Radiotherapy for Prostate Cancer: An End-to-End Phantom Study

The primary manuscript reports SF_IS, corresponding to the physical active chamber volume. SF_OS represents the complete chamber volume including the chamber walls and is provided here as a secondary sensitivity analysis. Triplicate measurements are reported to document within-session repeatability.

## Table S1. Repeatability of triplicate ion-chamber measurements

| Configuration | Plan | Location | Triplicate measurements [Gy] | | | Summary statistics | | | |
|---|---|---|---|---|---|---|---|---|---|
| | | | M1 | M2 | M3 | Mean [Gy] | SD [Gy] | CV [%] | Range [Gy] |
| Type A | Scheduled | CTV | 2.905 | 2.908 | 2.906 | 2.9063 | 0.0015 | 0.053 | 0.003 |
| Type A | Scheduled | Rectum | 1.672 | 1.673 | 1.671 | 1.6720 | 0.0010 | 0.060 | 0.002 |
| Type A | Scheduled | Bladder | 0.131 | 0.131 | 0.129 | 0.1303 | 0.0012 | 0.886 | 0.002 |
| Type B | Scheduled | CTV | 2.937 | 2.939 | 2.938 | 2.9380 | 0.0010 | 0.034 | 0.002 |
| Type B | Scheduled | Rectum | 1.488 | 1.491 | 1.491 | 1.4900 | 0.0017 | 0.116 | 0.003 |
| Type B | Scheduled | Bladder | 0.488 | 0.489 | 0.488 | 0.4883 | 0.0006 | 0.118 | 0.001 |
| Type A | Adapted | CTV | 2.918 | 2.917 | 2.919 | 2.9180 | 0.0010 | 0.034 | 0.002 |
| Type A | Adapted | Rectum | 1.442 | 1.441 | 1.441 | 1.4413 | 0.0006 | 0.040 | 0.001 |
| Type A | Adapted | Bladder | 0.131 | 0.130 | 0.130 | 0.1303 | 0.0006 | 0.443 | 0.001 |
| Type B | Adapted | CTV | 2.915 | 2.915 | 2.916 | 2.9153 | 0.0006 | 0.020 | 0.001 |
| Type B | Adapted | Rectum | 1.431 | 1.431 | 1.430 | 1.4307 | 0.0006 | 0.040 | 0.001 |
| Type B | Adapted | Bladder | 0.769 | 0.770 | 0.769 | 0.7693 | 0.0006 | 0.075 | 0.001 |
| A→B | Cross-adapted | CTV | 2.928 | 2.929 | 2.928 | 2.9283 | 0.0006 | 0.020 | 0.001 |
| A→B | Cross-adapted | Rectum | 1.285 | 1.285 | 1.284 | 1.2847 | 0.0006 | 0.045 | 0.001 |
| A→B | Cross-adapted | Bladder | 0.134 | 0.135 | 0.133 | 0.1340 | 0.0010 | 0.746 | 0.002 |
| B→A | Cross-adapted | CTV | 2.922 | 2.922 | 2.921 | 2.9217 | 0.0006 | 0.020 | 0.001 |
| B→A | Cross-adapted | Rectum | 1.570 | 1.570 | 1.568 | 1.5693 | 0.0012 | 0.074 | 0.002 |
| B→A | Cross-adapted | Bladder | 0.134 | 0.135 | 0.135 | 0.1347 | 0.0006 | 0.429 | 0.001 |

*Note: SD is the sample standard deviation ($n-1$; $n = 3$). These triplicates quantify within-session measurement repeatability and do not represent between-session reproducibility.*

## Table S2. SF_OS sensitivity analysis corresponding to scheduled/reference-plan measurements

| Configuration | Plan | Location | Dose [Gy] | | | Ethos vs measurement | | | Mobius vs measurement | |
|---|---|---|---|---|---|---|---|---|---|---|
| | | | Ethos SF_OS | Mobius SF_OS | Measured mean ± SD | Ethos local Δ [%] | Ethos ΔRx [%] | Ethos \|Δ\| [Gy] | Mobius local Δ [%] | Mobius ΔRx [%] |
| Type A | Scheduled | CTV | 2.910 | 2.936 | 2.9063 ± 0.0015 | -0.1 | -0.12 | 0.004 | -1.0 | -0.99 |
| Type A | Reference | CTV | 2.910 | 2.951 | 2.9063 ± 0.0015 | -0.1 | -0.12 | 0.004 | -1.5 | -1.51 |
| Type A | Scheduled | Rectum | 1.660 | 1.624 | 1.6720 ± 0.0010 | +0.7 | +0.41 | 0.012 | +3.0 | +1.61 |
| Type A | Reference | Rectum | 1.660 | 1.638 | 1.6720 ± 0.0010 | +0.7 | +0.41 | 0.012 | +2.1 | +1.14 |
| Type A | Scheduled | Bladder | 0.130 | 0.107 | 0.1303 ± 0.0012 | +0.3 | +0.01 | 0.000 | +21.9 | +0.78 |
| Type A | Reference | Bladder | 0.120 | 0.101 | 0.1303 ± 0.0012 | +8.7 | +0.35 | 0.010 | +28.8 | +0.97 |
| Type B | Scheduled | CTV | 2.910 | 2.934 | 2.9380 ± 0.0010 | +1.0 | +0.93 | 0.028 | +0.1 | +0.13 |
| Type B | Reference | CTV | 2.920 | 2.913 | 2.9380 ± 0.0010 | +0.6 | +0.60 | 0.018 | +0.8 | +0.82 |
| Type B | Scheduled | Rectum | 1.460 | 1.415 | 1.4900 ± 0.0017 | +2.0 | +0.99 | 0.030 | +5.2 | +2.48 |
| Type B | Reference | Rectum | 1.470 | 1.440 | 1.4900 ± 0.0017 | +1.3 | +0.65 | 0.020 | +3.4 | +1.64 |
| Type B | Scheduled | Bladder | 0.450 | 0.782 | 0.4883 ± 0.0006 | +8.4 | +1.26 | 0.038 | -37.6 | -9.79 |
| Type B | Reference | Bladder | 0.400 | 0.632 | 0.4883 ± 0.0006 | +21.9 | +2.92 | 0.088 | -22.8 | -4.80 |

## Table S3. SF_OS sensitivity analysis corresponding to adapted/reference-plan measurements

| Configuration | Plan | Location | Dose [Gy] | | | Ethos vs measurement | | | Mobius vs measurement | |
|---|---|---|---|---|---|---|---|---|---|---|
| | | | Ethos SF_OS | Mobius SF_OS | Measured mean ± SD | Ethos local Δ [%] | Ethos ΔRx [%] | Ethos \|Δ\| [Gy] | Mobius local Δ [%] | Mobius ΔRx [%] |
| Type A | Adapted | CTV | 2.910 | 2.927 | 2.9180 ± 0.0010 | +0.3 | +0.27 | 0.008 | -0.3 | -0.30 |
| Type A | Reference | CTV | 2.910 | 2.951 | 2.9180 ± 0.0010 | +0.3 | +0.27 | 0.008 | -1.1 | -1.11 |
| Type A | Adapted | Rectum | 1.420 | 1.386 | 1.4413 ± 0.0006 | +1.5 | +0.71 | 0.021 | +4.0 | +1.83 |
| Type A | Reference | Rectum | 1.490 | 1.485 | 1.4413 ± 0.0006 | -3.3 | -1.62 | 0.049 | -3.0 | -1.46 |
| Type A | Adapted | Bladder | 0.130 | 0.113 | 0.1303 ± 0.0006 | +0.3 | +0.01 | 0.000 | +15.1 | +0.57 |
| Type A | Reference | Bladder | 0.120 | 0.101 | 0.1303 ± 0.0006 | +8.7 | +0.35 | 0.010 | +28.8 | +0.97 |
| Type B | Adapted | CTV | 2.910 | 2.919 | 2.9153 ± 0.0006 | +0.2 | +0.17 | 0.005 | -0.1 | -0.13 |
| Type B | Reference | CTV | 2.910 | 2.939 | 2.9153 ± 0.0006 | +0.2 | +0.17 | 0.005 | -0.8 | -0.79 |
| Type B | Adapted | Rectum | 1.380 | 1.290 | 1.4307 ± 0.0006 | +3.6 | +1.66 | 0.050 | +10.8 | +4.66 |
| Type B | Reference | Rectum | 1.470 | 1.440 | 1.4307 ± 0.0006 | -2.7 | -1.34 | 0.040 | -0.7 | -0.35 |
| Type B | Adapted | Bladder | 0.800 | 1.047 | 0.7693 ± 0.0006 | -3.8 | -1.02 | 0.031 | -26.5 | -9.26 |
| Type B | Reference | Bladder | 0.600 | 0.632 | 0.7693 ± 0.0006 | +28.2 | +5.65 | 0.169 | +21.8 | +4.59 |

## Table S4. SF_OS sensitivity analysis corresponding to cross-evaluation measurements

| Configuration | Plan | Location | Dose [Gy] | | | Ethos vs measurement | | | Mobius vs measurement | |
|---|---|---|---|---|---|---|---|---|---|---|
| | | | Ethos SF_OS | Mobius SF_OS | Measured mean ± SD | Ethos local Δ [%] | Ethos ΔRx [%] | Ethos \|Δ\| [Gy] | Mobius local Δ [%] | Mobius ΔRx [%] |
| A→B | Cross-adapted | CTV | 2.910 | 2.937 | 2.9283 ± 0.0006 | +0.6 | +0.60 | 0.018 | -0.3 | -0.29 |
| A→B | Cross-scheduled | CTV | 2.910 | 2.939 | 2.9283 ± 0.0006 | +0.6 | +0.60 | 0.018 | -0.4 | -0.38 |
| A→B | Cross-adapted | Rectum | 1.330 | 1.350 | 1.2847 ± 0.0006 | -3.4 | -1.50 | 0.045 | -4.8 | -2.16 |
| A→B | Cross-scheduled | Rectum | 1.640 | 1.638 | 1.2847 ± 0.0006 | -21.6 | -11.83 | 0.355 | -21.6 | -11.77 |
| A→B | Cross-adapted | Bladder | 0.130 | 0.115 | 0.1340 ± 0.0010 | +3.4 | +0.15 | 0.004 | +16.7 | +0.64 |
| A→B | Cross-scheduled | Bladder | 0.119 | 0.107 | 0.1340 ± 0.0010 | +12.9 | +0.51 | 0.015 | +25.7 | +0.92 |
| B→A | Cross-adapted | CTV | 2.910 | 2.918 | 2.9217 ± 0.0006 | +0.4 | +0.40 | 0.012 | +0.1 | +0.12 |
| B→A | Cross-scheduled | CTV | 2.910 | 2.939 | 2.9217 ± 0.0006 | +0.4 | +0.40 | 0.012 | -0.6 | -0.55 |
| B→A | Cross-adapted | Rectum | 1.530 | 1.503 | 1.5693 ± 0.0012 | +2.6 | +1.31 | 0.039 | +4.4 | +2.20 |
| B→A | Cross-scheduled | Rectum | 1.470 | 1.440 | 1.5693 ± 0.0012 | +6.8 | +3.31 | 0.099 | +8.9 | +4.30 |
| B→A | Cross-adapted | Bladder | 0.130 | 0.115 | 0.1347 ± 0.0006 | +3.7 | +0.16 | 0.005 | +17.1 | +0.66 |
| B→A | Cross-scheduled | Bladder | 0.400 | 0.432 | 0.1347 ± 0.0006 | -66.3 | -8.84 | 0.265 | -68.8 | -9.89 |

*Note: Local difference = 100 × (Dmeas − Dcalc)/Dcalc; Rx-normalized difference = 100 × (Dmeas − Dcalc)/3.0 Gy; absolute difference = |Dmeas − Dcalc|. SF_OS is not the physical sensitive volume of the chamber and is presented only as a sensitivity analysis.*
